# Functionalized Hypercrosslinked High Internal Phase Emulsion Materials Based on 4-Vinylbenzyl Chloride for Anionic Dye Adsorption

**DOI:** 10.3390/ma19173680

**Published:** 2026-08-29

**Authors:** Eftychia Tsolka, Labrini Sygellou, Georgios Bokias, Valadoula Deimede

**Affiliations:** 1 Department of Chemistry, University of Patras, GR-26504 Patras, Greece; efi.tsolka@gmail.com (E.T.); bokias@upatras.gr (G.B.); 2 Foundation for Research and Technology—Hellas (FORTH), Institute of Chemical Engineering Science (ICE-HT), Stadiou St., GR-26504 Patras, Greece; sygellou@iceht.forth.gr

**Keywords:** PolyHIPEs, hypercrosslinking, Acid Blue 113, dye, adsorption, wastewater treatment

## Abstract

Synthesis and characterization of porous polymeric monoliths as high-performance adsorbents for the removal of Acid Blue 113 dye from aqueous solutions is reported. High internal phase emulsion (HIPE) polymerization process was employed for the synthesis of polymeric-HIPEs (polyHIPEs) using 4-vinylbenzyl chloride (VBC) and divinylbenzene (DVB) as co-monomers. To optimize their adsorption properties by inducing micro/meso porosity and introducing cationic functionalities, the polyHIPEs were post-modified via a two-step procedure. The first step involves the Friedel–Crafts hypercrosslinking reaction using FeCl_3_ as a catalyst, leading to high-surface-area materials, followed by cationic modification of residual chloromethyl groups with triethylamine to incorporate positively charged ammonium functionalities. The prepared polyHIPEs were characterized by Attenuated Total Reflectance-Fourier-Transform Infrared (ATR-FTIR), X-ray Photoelectron spectroscopy (XPS), Brunauer–Emmett–Teller surface area analysis (BET), and Scanning Electron Microscopy (SEM). BET analysis verified the huge surface area enhancement from 10.1 to 614.5 m^2^ g^−1^ due to hypercrosslinking. The modified cationic adsorbent showed higher adsorption capacity (280.1 mg g^−1^) compared to the non-functionalized analogue (110 mg g^−1^) for anionic dye Acid Blue 113. Adsorption followed pseudo-second-order kinetics, suggesting chemisorption as the dominant mechanism. The adsorption mechanism was investigated via XPS, revealing that electrostatic interactions along with hydrogen bonding and π-π interactions are major contributors to this process.

## 1. Introduction

The globalization of industrial operations–particularly textile, leather, and paper processing—has resulted in the respective release of high concentrations of artificial dyes into natural water circuits. Dye contamination of water bodies reduces sunlight penetration, thereby impairing photosynthesis and inhibiting plant growth. In addition, serious environmental and public health concerns have been raised owing to their carcinogenic and mutagenic properties. Despite advances in developing innovative, sustainable technologies for effective dye removal from wastewater, it remains a key environmental and technological issue due to limitations of conventional treatment technologies (e.g., inefficiency, high cost, and secondary pollution potential) [1,2,3]. Adsorption is recognized as one of the most efficient and cost-effective technologies for dye removal from wastewater, as it relies on the potential for absorbent regeneration and recycling. However, the high production cost of some high-capacity adsorbents is a drawback despite the overall efficiency of the technique. This limitation can be overcome by utilizing cheap raw materials as alternative adsorbents, thereby offering a sustainable and economically viable option [4].

Polymeric materials and particularly macroporous polymers in monolithic form (known as polyHIPEs) [5,6] can be easily produced by a simple polymerization method of a continuous phase of high internal phase emulsion (HIPE). HIPEs are emulsions where the volume fraction of the internal phase is greater than 0.74 of the total volume consisting of both the internal and continuous phases [6,7,8]. For example, the most common hydrophobic polyHIPE system can be prepared using a water-in-oil (w/o) HIPE in which hydrophobic monomers such as styrene (ST), 4-vinylbenzyl chloride (VBC), and divinylbenzene (DVB) are used as the continuous phase for polymerization [8]. From a morphological perspective, the macroporous polyHIPEs are characterized by their distinctive “voids” created by the extraction of water droplets (1–30 μm), that are interconnected through “windows” (0.5 to 3 μm) [6]. These macroporous structures display unique properties such as open-cellular morphology, porosity with excellent interconnectivity, good thermal and mechanical stability, easy and low-cost synthesis, scale-up, and easy surface functionalization [6,8], enabling their use in separation, catalysis, and adsorption applications [9,10,11,12,13,14,15,16,17]. Hydrophilic polyHIPEs bearing ionic or ionizable functionalities were recently successfully applied for dye adsorption as well [14,15,16,17,18]. However, due to their low surface areas (3−10 m^2^ g^−1^), the adsorption kinetics are slow. Therefore, increasing surface area via introduction of micro/mesopores is essential for the development of an ideal adsorbent for the removal of dyes.

One strategy to enhance the surface area of polyHIPEs is hypercrosslinking via Friedel−Crafts alkylation [19,20]. For example, hypercrosslinked polyHIPEs based on VBC have been successfully prepared via Friedel–Crafts-catalyzed transformation of chloromethyl groups of VBC into crosslinking methylene bridges using FeCl_3_ as catalyst. Prior to crosslinking, the precursor polyHIPEs must swell in a thermodynamically good solvent to achieve a homogeneous cross-linking distribution throughout the rigid polymeric network, thereby preventing pore collapse after solvent removal [21,22]. The new micropore formation during cross-linking results in extremely high surface areas (as high as 2000 m^2^ g^−1^) [23,24]. This method does not affect the emulsion-templated macroporosity of polyHIPEs that ensures fast mass transfer, while the induced microporosity, enabling greater accessibility, could be controlled by varying the concentration of DVB cross-linker during polyHIPE formation [23,24,25]. Through this simple, low-cost method, hierarchically porous, high-surface-area polyHIPEs, which possess uniform micro-mesopore distribution ranging from 0.5 to 20 nm, can be synthesized and applied in diverse applications including adsorption [26,27,28,29,30,31,32]. For example, Suslukaya et al. reported the synthesis of sulphonamide functionalized hypercrosslinked VBC/DVB monoliths through emulsion templating. The hypercrosslinking reaction enables the introduction of meso- and microporosities to the interconnected framework of hierarchically macroporous polyHIPE (specific surface areas up to 970 m^2^ g^−1^) while the sulfonamide functional groups facilitate the selective interaction with human serum albumin [29]. These materials exhibited rapid kinetics and selective adsorption towards albumin macromolecules because of their high surface area with surface functionality design. Similarly, Uzum et al. prepared carboxylic acid-functionalized hypercrosslinked VBC/DVB porous polymers for methylene blue removal [30]. Through control of the emulsion parameters and optimization of the reaction time of hypercrosslinking in terms of adsorption kinetics and adsorption capacity, hypercrosslinked polyHIPEs with BET surface areas greater than 600 m^2^ g^−1^, high adsorption capacity (maximum adsorption capacity of 450 m^2^ g^−1^), and fast uptake for the cationic dye methylene blue were achieved. The hypercrosslinked polyHIPE could also be reused after desorption with acid treatment, which is an advantage of these materials. In another study, Castaldo et al. prepared amino-functionalized hypercrosslinked VBC/DVB polyHIPE resins with BET surface areas ranging from 86 to 180 m^2^/g and high sorption capacity for volatile organic compounds and CO_2_ [32]. Very recently, the synthesis of positively charged hydrophobic polymer foams via water-in-oil (W/O) HIPE polymerization followed by quaternization, employing 4-vinylbenzyl chloride (VBC) and 4-tert-butylstyrene (tBS) as the comonomers and DVB as cross-linker for adsorptive emulsion separation was reported [33].

Based on our previous experience on the synthesis of polyHIPEs [14] as well as on VBC-containing materials [34,35], the preparation of cationic functionalized, high surface area, hierarchically porous polyHIPE adsorbents for efficient anionic dye removal is reported in the present work. Initially, the hydrophobic VBC/DVB-based polyHIPE precursor was prepared from a water-in-oil (w/o) high internal phase emulsion (HIPE), using 4-VBC and DVB as monomer and cross-linking agent, respectively. Next, targeting hypercrosslinking and quaternization, the Friedel–Crafts alkylation reaction was applied to induce micropores, which resulted in high surface area polyHIPEs, followed by cationic modification of the remaining methylene chloride functional groups with triethylamine (TEAM). The as-prepared cationic functionalized material was fully characterized in terms of structure, morphology, and adsorption performance (isotherm and kinetic studies) against the anionic dye Acid Blue 113.

## 2. Materials and Methods

### 2.1. Materials

All chemicals and solvents used for the synthesis of the adsorbents were of analytical grade and used without any prior treatment. 4-vinylbenzyl chloride (4-VBC, 90%), divinylbenzene (DVB, 80%), sorbitan monooleate (Span 80), calcium chloride (CaCl_2_, ≥93%), ferric chloride (FeCl_3_, 99%) potassium persulfate (KPS, 99%), triethylamine (TEAM, ≥99.5%), acid blue 113 (molecular formula: C_32_H_21_N_5_Na_2_O_6_S_2_; molecular weight: 681.65 g/mol, used as a model water soluble anionic dye) were purchased from Sigma-Aldrich (St. Louis, MO, USA). Ethanol (99%), dimethylformamide (DMF), 1,2-dichloroethane (DCE), methanol, and acetone were supplied by Carlo Erba (Milan, Italy). Ultrapure water was produced through an Arium mini water purification system (Sartorius, Göttingen, Germany).

### 2.2. Methods

#### 2.2.1. Synthesis of PolyHIPE Precursors (polyHIPE P(VBC-co-DVB_20_))

A typical water-in-oil (w/o) high internal phase emulsion (HIPE) with a 90% vol aqueous internal phase was prepared as follows. 4-VBC (4.3g, 0.028 mol) was added to a reactor along with divinylbenzene (0.9 g, 0.007 mol) and the surfactant Span 80 (1.5 g). The mixture was stirred at 350 rpm. The calcium chloride (CaCl_2_, 0.5 g) and the initiator potassium persulfate (KPS, 0.1 g) were dissolved in deionized water, adjusting the total volume of the aqueous phase to 45 mL. The aqueous mixture was then added dropwise into the organic phase under continuous stirring at 500 rpm. Once the addition was complete, stirring was maintained for 20 min for the formation of a homogeneous creamy water-in-oil emulsion. The emulsion was transferred to a Falcon tube and polymerized in a water bath at 65 °C for 24 h. The synthesized monolith was cut into discs (with a thickness of 0.5–1 cm) and Soxhlet-extracted with ethanol for 24 h to remove the unreacted components. Finally, the polyHIPE material was vacuum-dried at 50 °C for at least 24 h. The experimental conditions used for the preparation of polyHIPEs are given in Table 1.

#### 2.2.2. Hypercrosslinking of PolyHIPE Precursors (H-P(VBC-co-DVB_20_))

The polyHIPE monolith was ground into a powder prior to hypercrosslinking to ensure a uniform reaction. PolyHIPE powder (2 g) was introduced into a round-bottom flask, followed by the addition of 100 mL of 1,2-dichloroethane. The flask was sealed with a rubber septum, and the mixture was degassed by purging with a continuous stream of argon for 15 min under constant stirring. Then, the sealed flask was stirred for 24 h to allow the polymer to swell. Subsequently, the flask was placed in an ice bath, FeCl_3_ (catalyst) was added, and the mixture was purged again with argon for 15 min and left for an additional 45 min under stirring to ensure homogeneous dispersion of FeCl_3_. The flask was then transferred to an oil bath and heated at 80 °C for 30 min, 60 min, or 180 min. After the specified reaction time, the reaction was immediately quenched by adding 40 mL of excess methanol. Following filtration to isolate the hypercrosslinked polymer, the obtained solid was washed multiple times with methanol (3 × 20 mL). The unreacted reagents or impurities were removed by extraction with acetone in a Soxhlet extractor (Quickfit, England) for 24 h, followed by vacuum-drying at 45 °C for at least 24 h.

#### 2.2.3. Cationic Functionalization of Hypercrosslinked PolyHIPE with Triethylamine (H-P(VBCTEAM-co-DVB_20_))

To a degassed 250 mL round-bottom flask, purged with argon, fitted with a condenser and a magnetic stirrer, 0.8 g (5.4 mmol) of hypercrosslinked polyHIPE was added and allowed to swell in 80 mL dimethylformamide (DMF). Triethylamine (1.81 mL, 12.9 mmol) was then introduced into the mixture. The reaction mixture was stirred and heated at 80 °C for 24 h. Following completion of the reaction, the solid product was filtered and washed sequentially with DMF and ethanol. Finally, the solid was dried in a vacuum oven at 60 °C for 12 h.

### 2.3. Characterization

Attenuated Total Reflectance Fourier Transform Infrared (ATR FT-IR) spectra were collected using an Alpha II spectrometer (Brucker, Billerica, MA, USA) with a Platinum diamond ATR module. All spectra were recorded in the range of 4000–400 cm^−1^ with an average of 34 scans and a spectral resolution of 4 cm^−1^. Thermogravimetric analysis (TGA) curves were recorded by employing a Labsys TG, Setaram Instrumentation (Caluire-et-Cuire, France) over the temperature range of 25 to 800 °C at a heating rate of 20 °C min^−1^ under a nitrogen atmosphere. Before testing, the samples were dried at 80 °C for 24 h to remove residual moisture and solvents. The BET specific surface area (SBET) was determined via nitrogen adsorption–desorption at 77K (p/p_0_: 0.02–0.3) using a Micromeritics Gemini 2375 instrument (Micromeritics Instrument Corporation, Norcross, GA, USA), averaging two measurements for accuracy.

(1) $$P_{x}=\frac{V_{x}}{V_{total}}\times 100,$$

where $P_{x}$ represents the percentage (%) of macropores, mesopores, and micropores, respectively, out of the total volume, $V_{x}$ represents the volume of each respective pore size, and $V_{total}$ denotes the total pore volume. Specifically, the pore size ranges were classified as macropores for pore widths ≥ 50 nm, mesopores from 2 to 50 nm, and micropores for pore widths ≤ 2 nm.

SEM images with a 5.00 kX magnification were recorded on a JEOL JSM 6300 instrument (JEOL, Akishima, Tokyo, Japan) equipped with an X-ray energy dispersive spectrometer, EDS (ISIS Link 300, Oxford Instruments, High Wycombe, UK). Dry powder samples were mounted on aluminum SEM stubs using double-sided conductive carbon tape and coated with a conductive Au film through sputtering. An ultra-high vacuum (UHV) system (with a background pressure in the range of 10^−11^–10^−7^ mbar) equipped with a SPECS Phoibos 100 1D-DLD hemispherical analyser (SPECS GmbH, Berlin, Germany) and a dual-anode Mg/Al X-ray source was used to conduct X-ray photon electron spectroscopy (XPS) experiments. The spectra were recorded with an Mg Kα X-ray source (1253.6 eV), and the data were processed using Specs Lab Prodigy software. An unmonochromatized Mg Kα line at 1253.6 eV and an analyzer pass energy of 15eV (giving a full width at half maximum (FWHM) of 0.85 eV for the Ag3d5/2 peak) was employed. The XPS core level spectra were analyzed using a fitting routine, which can decompose each spectrum into individual mixed Gaussian–Lorentzian peaks after a Shirley background subtraction.

### 2.4. Adsorption Kinetics and Equilibrium Experiments

To evaluate the adsorption capacity of porous polyHIPE monolithic structures, acid blue 113 dye was used as a model organic dye. Different parameters were systematically investigated, including the effect of adsorbent dosage (0.05–5 g), contact time, and initial pollutant concentration (10 to 100 mg L^−1^) on adsorption efficiency. For the batch experiments, specific amounts of adsorbents were added to glass vials containing 10 mL of dye solution. Throughout the process, the mixture was continuously stirred under a constant temperature of 25 °C. Following equilibration, the supernatant was analyzed by recording UV-VIS spectra (200–800 nm) using a Hitachi U-2900 UV-Vis spectrophotometer (Dallas, TX, USA). This enabled the determination of the residual Acid Blue 113 concentration at its maximum absorption peak (566 nm) (Figure S1).

The dye removal efficiency $R\left(\%\right)$, the amount of adsorbed dye at time t, $q_{t}$ (mg g^−1^) and the adsorption capacity at equilibrium $q_{e}$ (mg g^−1^) are the key parameters studied and are calculated by the following equations [36]:

(2) $$R\left(\%\right)=\left(\frac{C_{0}-C_{e}}{C_{0}}\right)\times 100,$$

(3) $$q_{t}=\left(\frac{C_{0}-C_{t}}{m}\right)V,$$

(4) $$q_{e}=\left(\frac{C_{0}-C_{e}}{m}\right)V,$$

where $C_{0}$, $C_{e}$, and $C_{t}$ (mg L^−1^) are the initial dye concentrations, at equilibrium and at time t, respectively, m (g) is the mass of the adsorbent, and V (L) is the volume of the dye solution. Two replicates were used for each sample.

The dye adsorption kinetics on polyHIPEs were studied by employing Lagergren pseudo-first-order (Equation (5)) [36] or pseudo-second-order (Equation (6)) [37] models, as given below:

(5) $$\ln\left(q_{e}-q_{t}\right)=ln(q_{e})-k_{1}t,$$

(6) $$\frac{1}{q_{t}}=\frac{1}{k_{2}q_{e}^{2}}+\frac{1}{q_{e}}t,$$

where $k_{1}$ (1 min^−1^) and $k_{2}$ (g mg^−1^ min^−1^) are the rate constants of the pseudo-first-order and pseudo-second-order models, respectively.

The dye adsorption equilibrium data on polyHIPEs were correlated to the Langmuir and Freundlich adsorption isotherms. The Langmuir model is as follows [38]:

(7) $$\frac{C_{e}}{q_{e}}=\frac{C_{e}}{q}+\frac{1}{K_{L}q}$$

where q (mg g^−1^) corresponds to the maximum adsorption capacity and $K_{L}$ (L mg^−1^) is the Langmuir adsorption constant.

The dimensionless constant separation factor, R_L_, is given by the following equation:

(8) $$R_{L}=\frac{1}{1+K_{L}C_{0}},$$

when 0 < $R_{L}$< 1, adsorption is favourable, and when $R_{L}$ > 1, it is unfavourable.

The Freundlich model is described by the following equation [38]:

(9) $$logq_{e}=logK_{F}+\frac{1}{n}\left(logC_{e}\right),$$

where $K_{F}$ (L mg^−1^) is the adsorption constant in the Freundlich isotherm model and $\frac{1}{n}$ when ranges between 0.1 and 1.0, indicates favourable adsorption conditions.

## 3. Results and Discussion

### 3.1. Synthesis and Characterizations of P(VBC-co-DVB_20_), H-P(VBC-co-DVB_20_) and H-P(VBCTEAM-co-DVB_20_)

**Figure 1:**
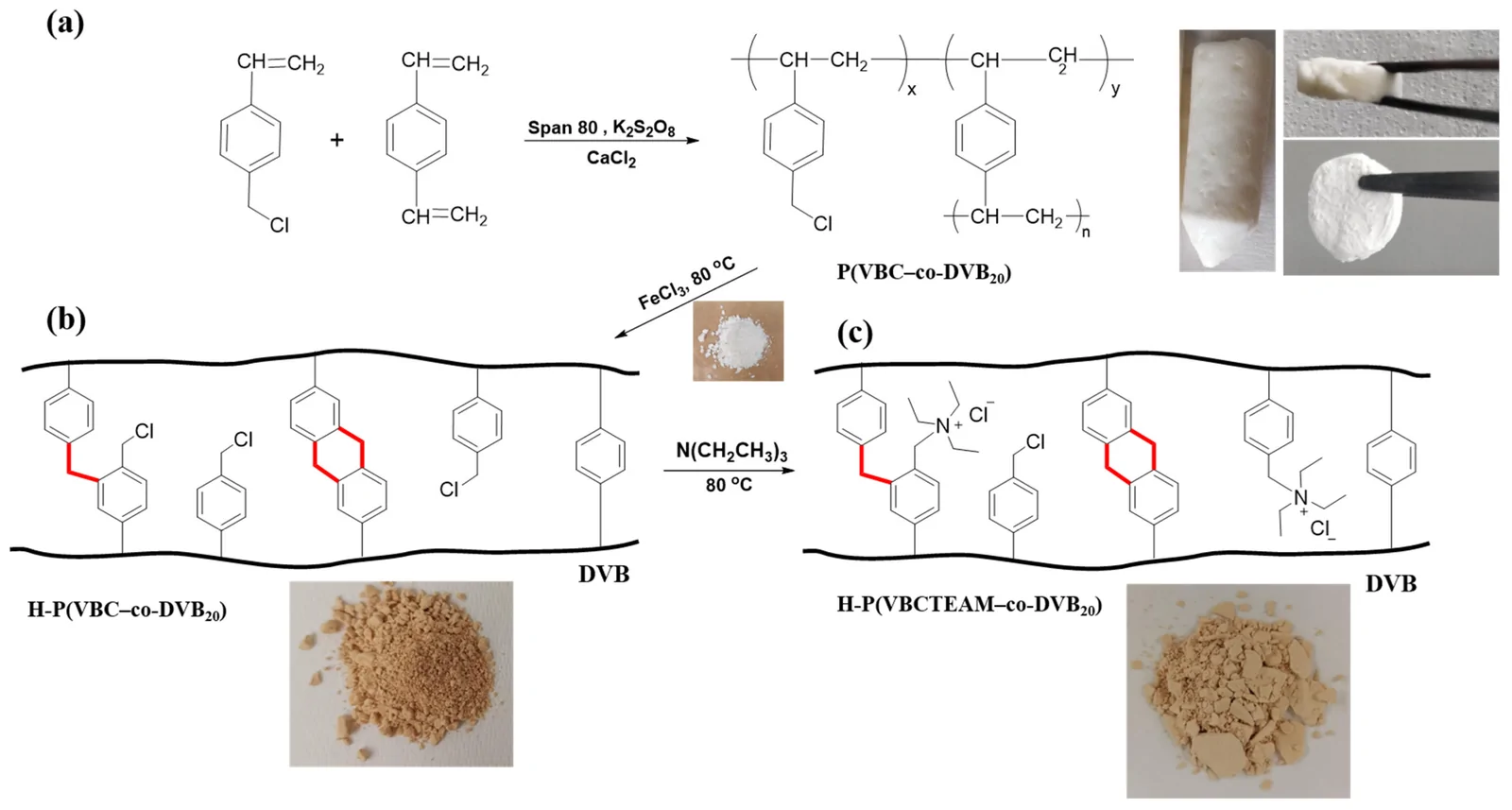
Synthetic route followed for the synthesis of (**a**) polyHIPE P(VBC-co-DVB_20_), (**b**) hypercrosslinked H-P(VBC-co-DVB_20_), and (**c**) ammonium functionalized analogue H-P(VBCTEAM-co-DVB_20_).

PolyHIPEs have recently been employed as adsorbents for the efficient removal of dyes from aqueous solutions. In particular, the increased adsorption capacity towards ionic dyes is related to the presence of ionic functionalities in their structure and microporosity as well [4,30]. To this end, macroporous polyHIPEs were synthesized as precursor materials followed by a two-step post-polymerization modification: hypercrosslinking reaction to induce micropores/mesopores and quaternization reaction to introduce cationic functionalities, thus resulting in ammonium-functionalized, high-surface-area, hierarchically porous analogues. First, the macroporous poly (4-vinylbenzyl chloride-divinylbenzene) based polyHIPE precursor (polyHIPE P(VBC-co-DVB_20_) was successfully synthesized via free radical polymerization of VBC and DVB initiated by KPS, using the high internal phase emulsion method, as described in the literature [8,37] (Figure 1). With the addition of 2.9 wt% surfactant Span 80 (Hydrophilic-Lipophilic Balance (HLB) = 4.3) from the oleate family of surfactants, which is suitable for reducing the interfacial tension in w/o emulsions, stable HIPE and polyHIPE monoliths were obtained [39] (Figure 1).

Next, the hypercrosslinking method was chosen as a simple method to induce microporosity, thus enabling the formation of a densely, highly crosslinked interconnected network with a microporous structure (H-P(VBC-co-DVB_20_)). Specifically, the PolyHIPE precursor monolith was ground into a powder form, left to swell in dichloroethane, and was hypercrosslinked through a Friedel–Crafts alkylation reaction catalyzed by a Lewis acid, FeCl_3_. Hypercrosslinking is a controlled reaction enabling the increase in the surface area in a controlled way due to the remaining unreacted pendant groups. Our primary goal was to preserve a small fraction of unreacted CH_2_-Cl functional groups of VBC units for their subsequent conversion to their cationic analogues. For this reason, the reaction time for Friedel–Crafts alkylation was limited to 30 min, 1 h, and 3 h so that the macroporous polyHIPE would gain hierarchical porosity through the formation of micro and mesopores while retaining the CH_2_-Cl functionality on its surface.

During the hypercrosslinking reaction, chloromethyl functional groups of VBC act as internal electrophiles and react with benzene rings, leading to the formation of methylene bridges (Figure 1). Furthermore, the formation of a six-membered ring between two benzyl chloride groups (self-linkage) takes place via a polycondensation reaction as well, which is reported as a characteristic of the hypercrosslinking of VBC-based polymeric precursors (Figure 1) [24,25,40]. The appearance of the material changes from white to yellow-brown (Figure 1 inset) after hypercrosslinking, which is attributed to FeCl_3_ [28].

**Figure 2:**
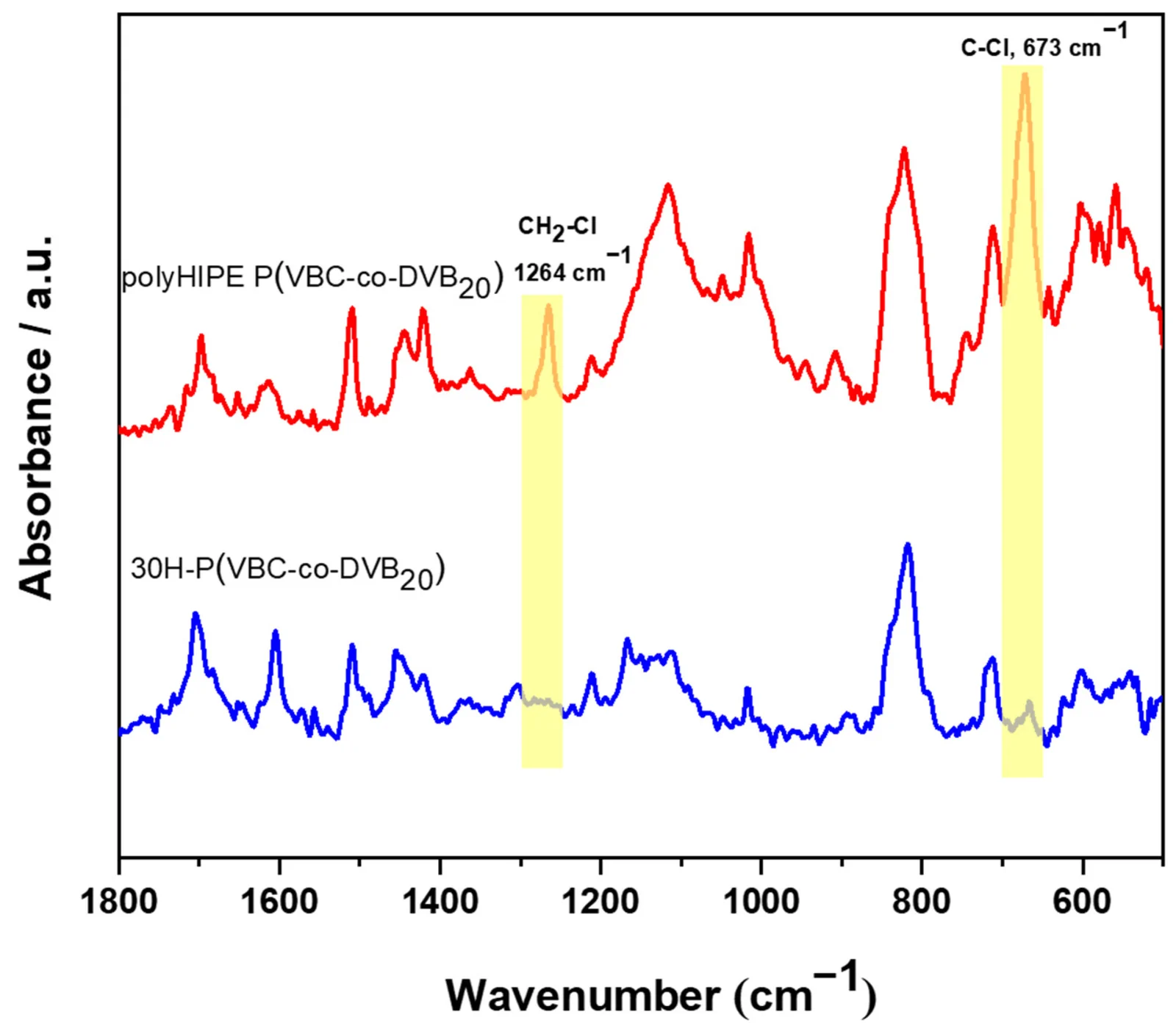
ATR—FTIR spectra of the prepared polyHIPE P(VBC-co-DVB_20_) and 30H-P(VBC-co-DVB_20_).

The ATR–FTIR spectra of the prepared polyHIPE (P(VBC-co-DVB_20_) and its analogue after 30 min hypercrosslinking (30H-P(VBC-co-DVB_20_)) are given in Figure 2. The intensity of the peaks at 673 cm^−1^ and 1264 cm^−1^ corresponding to C−Cl stretching and CH_2_-Cl wagging vibrations of VBC groups, respectively, was significantly decreased after the Friedel−Crafts hypercrosslinking reaction due to partial conversion of chloromethyl groups into crosslinking methylene bridges. In addition, some minor peaks at 744 cm^−1^ (unreacted phenyl rings) and 812 cm^−1^ (para-substituted benzene ring) attributed to VBC and DVB monomers were weakened or almost disappeared since hypercrosslinking and nucleophilic substitution reactions were successfully achieved.

To investigate the morphology, surface area, and porosity, analysis based on N_2_ adsorption/desorption isotherms was conducted for polyHIPE P(VBC-co-DVB_20_) and 30, 60, and 180 min hypercrosslinked polyHIPEs. The data derived from this analysis, including BET surface area (S_BET_), total pore volume, and average pore volume, are given in Table 2. As can be noted from Table 2, before the hypercrosslinking reaction, the polyHIPE P(VBC-co-DVB_20_) exhibited a relatively low specific surface area of 10.1 m^2^ g^−1^, owing to the macro pores created by emulsification of the monomer mixture, which is in line with literature [14]. The surface area was remarkably increased within only 30 min of hypercrosslinking reaction (614.5 m^2^ g^−1^), while further increase in reaction time resulted in lower specific area values (534.7 m^2^ g^−1^ and 461.6 m^2^ g^−1^ for reaction times of 60 min and 180 min, respectively). In addition, the prolonged reaction time resulted in reduced pore volume. Similar surface-specific area values after 15 and 30 min hypercrosslinking (603.3 and 757.66 m^2^ g^−1^, respectively) were also obtained for systems based on VBC polyHIPE (80 mol% VBC) reported in the literature [24].

**Figure 3:**
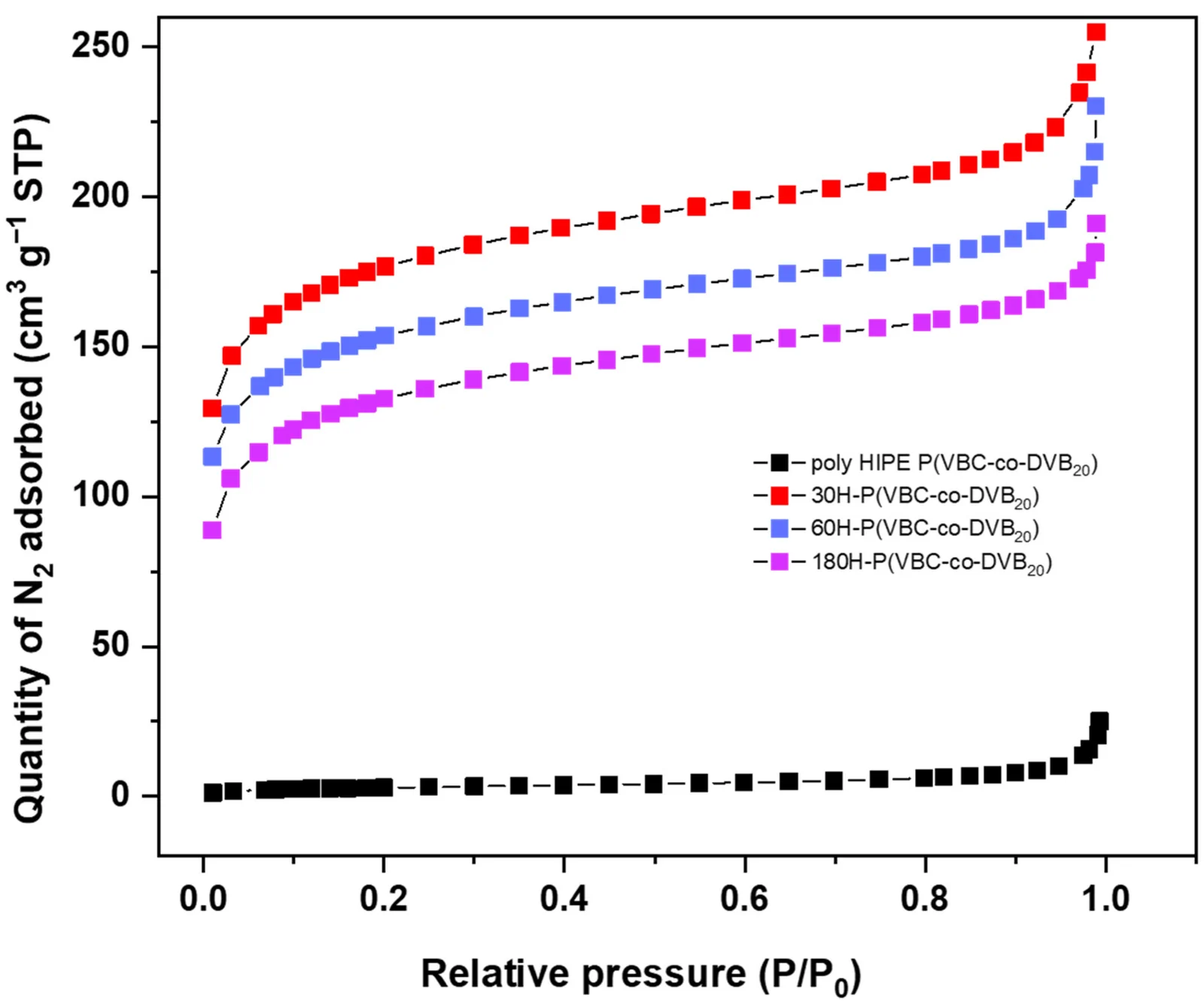
N_2_ adsorption isotherms of P(VBC-co-DVB_20_) and 30, 60 and 180 min hypercrosslinked polyHIPEs.

**Figure 4:**
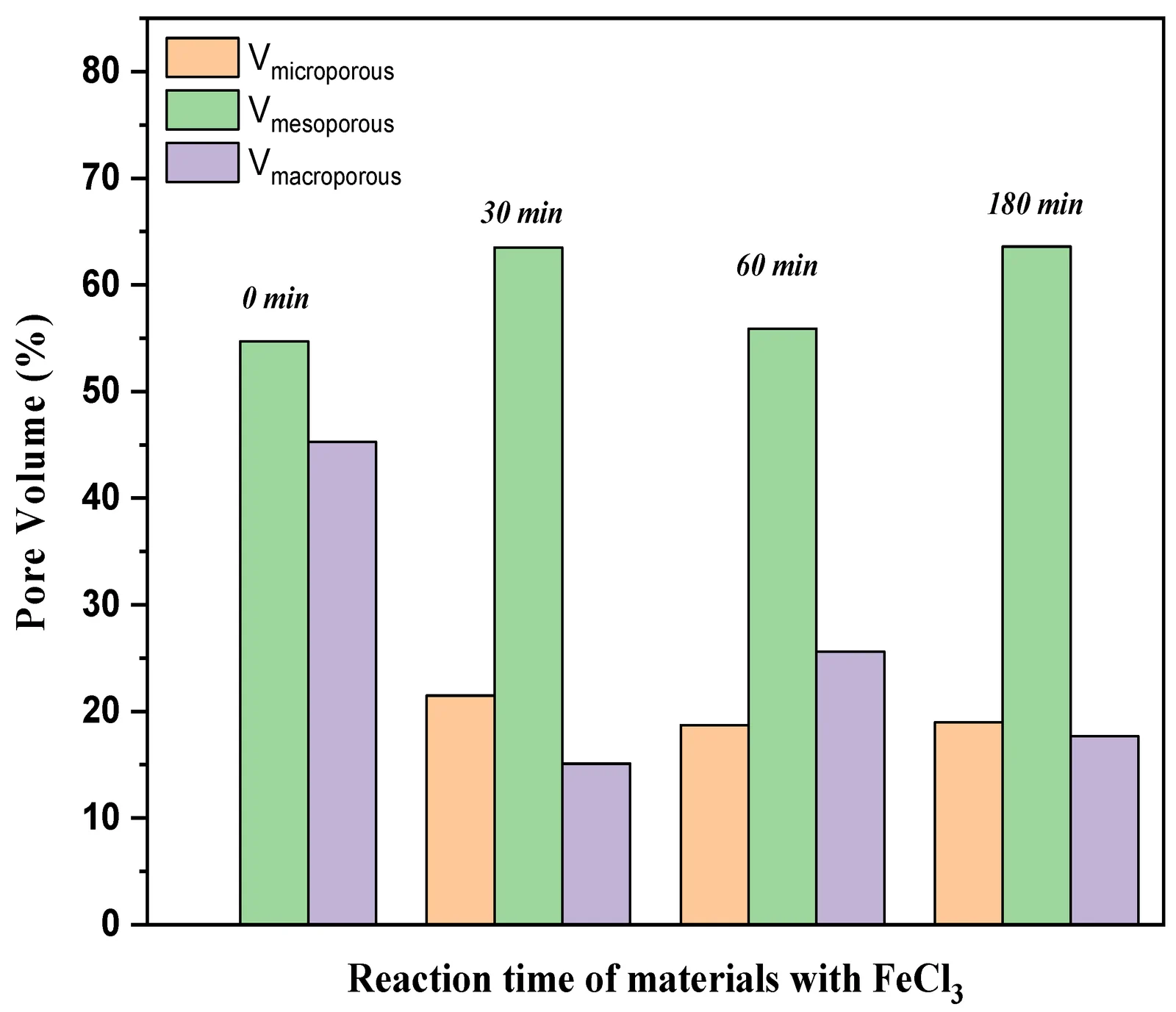
Effect of hypercrosslinking time on the pore distribution of P(VB-co-DVB_20_).

As can be observed from the nitrogen adsorption isotherms (Figure 3), the polyHIPE precursor exhibits a small adsorption of nitrogen. On the other hand, the nitrogen adsorption isotherm at a low relative pressure (P/P_0_ < 0.2) increases sharply after hypercrosslinking, indicating the presence of permanent micropores in all hypercrosslinked polyHIPE samples. In other words, the substantially higher BET surface of the hypercrosslinked polymers indicates the formation of new porosity [24,25] (Figure 4). This contribution of individual pore sizes was calculated using Equation (1). The increased microporosity is the result of the formation of an additional large number of methylene bridging units between the benzene rings, which are uniformly distributed throughout the network, thus preventing the shrinkage of the polymer chains and consequently enabling the formation of some new voids between chains. Based on the above data, the 30 min hypercrosslinked polyHIPE was chosen to be further studied for conversion to its cationic analogue due to its higher surface area promoting mass transfer and accessibility to the active sites.

**Figure 5:**
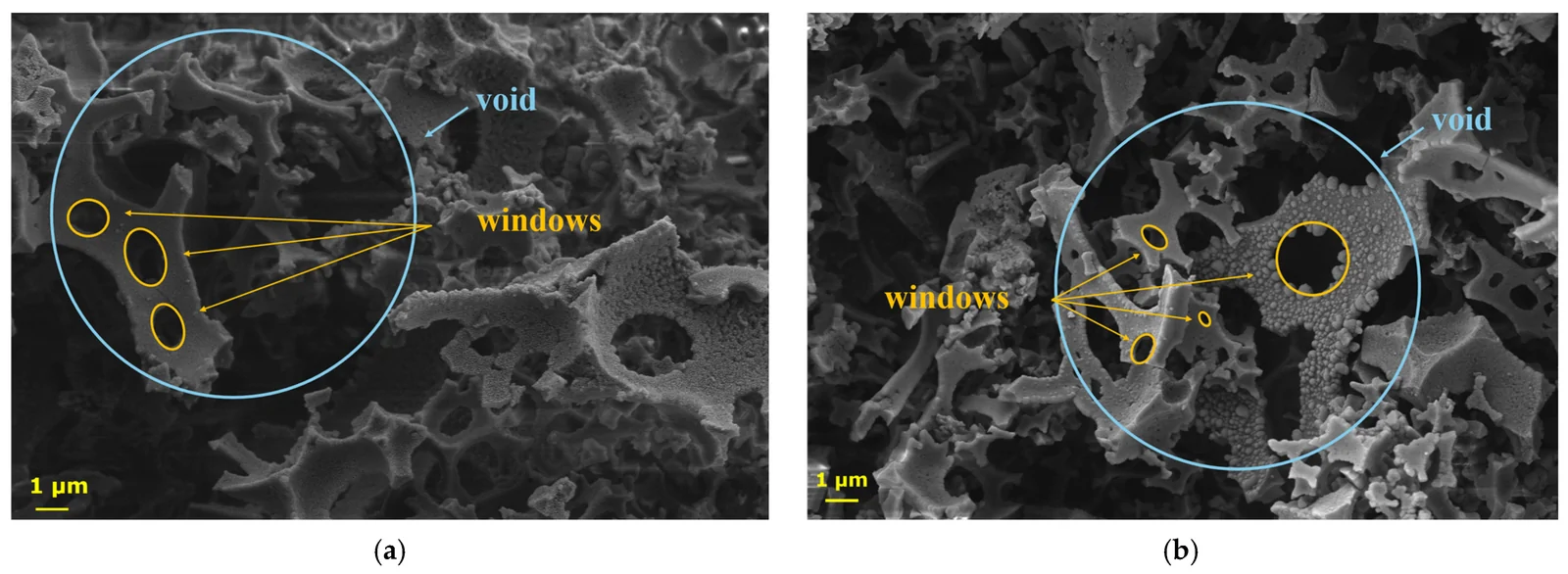
Cross-sectional SEM micrographs of the prepared (**a**) 30H-P(VBC-co-DVB_20_) and (**b**) cationic 30H-P(VBCTEAM-co-DVB_20_).

The macroporous structure of polyHIPE is preserved after crosslinking, as clearly observed in the cross-sectional SEM micrograph (Figure 5a) of 30H-P(VBC-co-DVB_20_). The characteristic morphology of polyHIPE observed consists of large spherical macropores interconnected by smaller windows with diameters ranging from 0.96 to 2.1 μm. The interconnected macropore morphology indicates the formation of a highly rigid network, which is consistent with previous reports [24,25].

Since the prepared hypercrosslinked polyHIPEs are basically hydrophobic, they have poor affinity to water; therefore, the introduction of cationic functionalities would induce hydrophilicity, enabling their use as high-performance polymeric adsorbents towards anionic dyes. For this reason, the hypercrosslinked polyHIPE 30H-P(VBC-co-DVB_20_) was further modified via reaction of remaining CH_2_-Cl groups of VBC with triethylamine, resulting in ammonium-functionalized high-surface polymeric adsorbents. As shown in Table 2, a small reduction in specific surface area was observed after quaternization, while the average pore size was significantly increased. In addition, from the SEM micrograph (Figure 5b) it is evident that the 30H-P(VBCTEAM-co-DVB_20_) still displays the characteristic macropore morphology interconnected with windows of diameter ranging between 0.12 and 0.65 μm.

**Figure 6:**
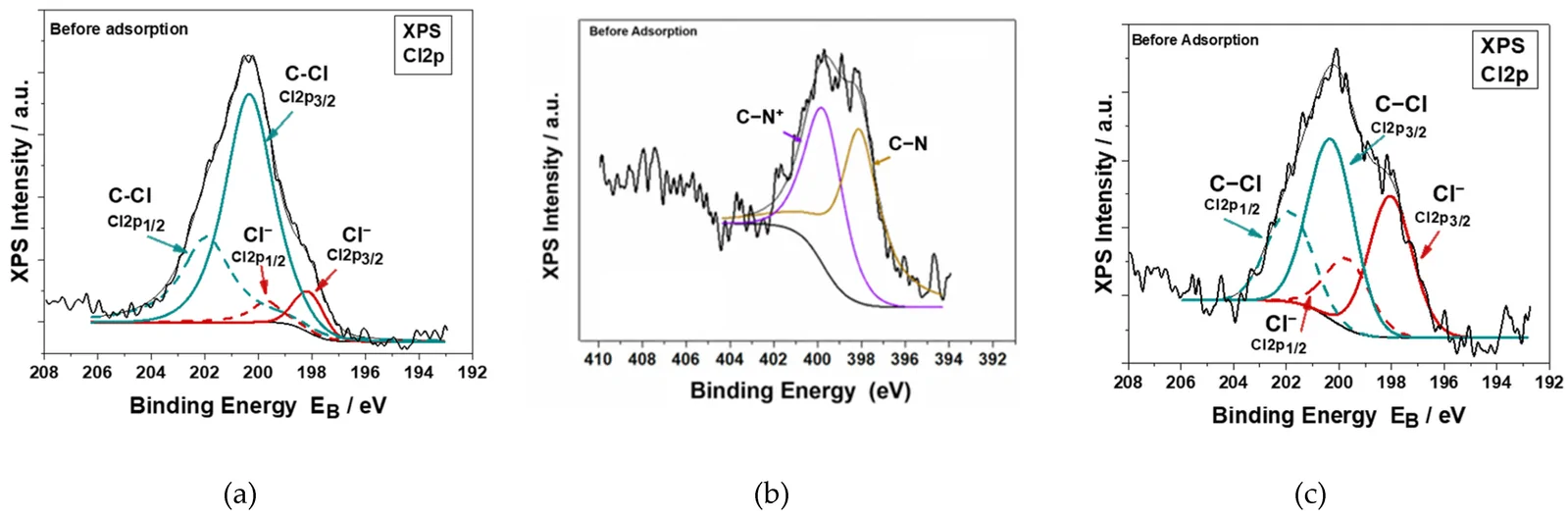
XPS spectra of the prepared (**a**) 30H-P(VBC-co-DVB_20_) and (**b**,**c**) cationic 30H-P(VBCTEAM-co-DVB_20_) as adsorbents before adsorption of Acid Blue 113 dye.

The surface chemistry of the hypercrosslinked polyHIPE 30H-P(VBC-co-DVB_20_) and its cationic analogue 30H-P(VBCTEAM-co-DVB_20_) was investigated via XPS. First, regarding the hypercrosslinked 30H-P(VBC-co-DVB_20_), the Cl2p core-level spectrum (Figure 6a) was curve-fitted into two main doublets (Cl2p3/2 and Cl2p1/2). The predominant doublet with the Cl2p3/2 peak located at 200.3 eV is attributed to neutral chlorine of the remaining chloromethyl groups (-CH_2_Cl) of the VBC units. Additionally, a minor doublet with a Cl2p3/2 peak at 197.9 eV is assigned to chloride anion (Cl^−^), which could be attributed to trace amounts of residual FeCl_3_ catalyst entrapped within the dense hypercrosslinked network. Subsequently, the surface chemistry of the cationic analogue, 30H-P(VBCTEAM-co-DVB_20_), was evaluated. The XPS core-level N1s and Cl2p spectra of the cationic polyHIPE are presented in Figure 6b,c, respectively. The XPS N1s core-level spectrum (Figure 6b) is curve-fitted into two peaks. The dominant peak at 401.2 eV is ascribed to the positively charged nitrogen (-N^+^(CH_2_CH_3_)_3_), which is consistent with the presence of ammonium groups in polyHIPE- structure [14,15], while the second one at 398.1 eV corresponds to C-N groups suggesting that some amount of triethylamine reagent is still present. In the XPS Cl2p core-level spectrum (Figure 6c), a Cl2p3/2 peak was observed at 197.7 eV, which corresponds to Cl^−^, while a second one at 200.3 eV, is attributed to the neutral chlorine of the remaining -CH_2_Cl groups in the 30H-P(VBCTEAM-co-DVB_20_). These data confirm the successful quaternization reaction. However, the presence of the peak at 200.3 eV indicates that partial conversion of chloromethyl groups to their cationic analogues took place. This is probably due to limited access of triethylamine to active sites owing to its bulkiness.

**Figure 7:**
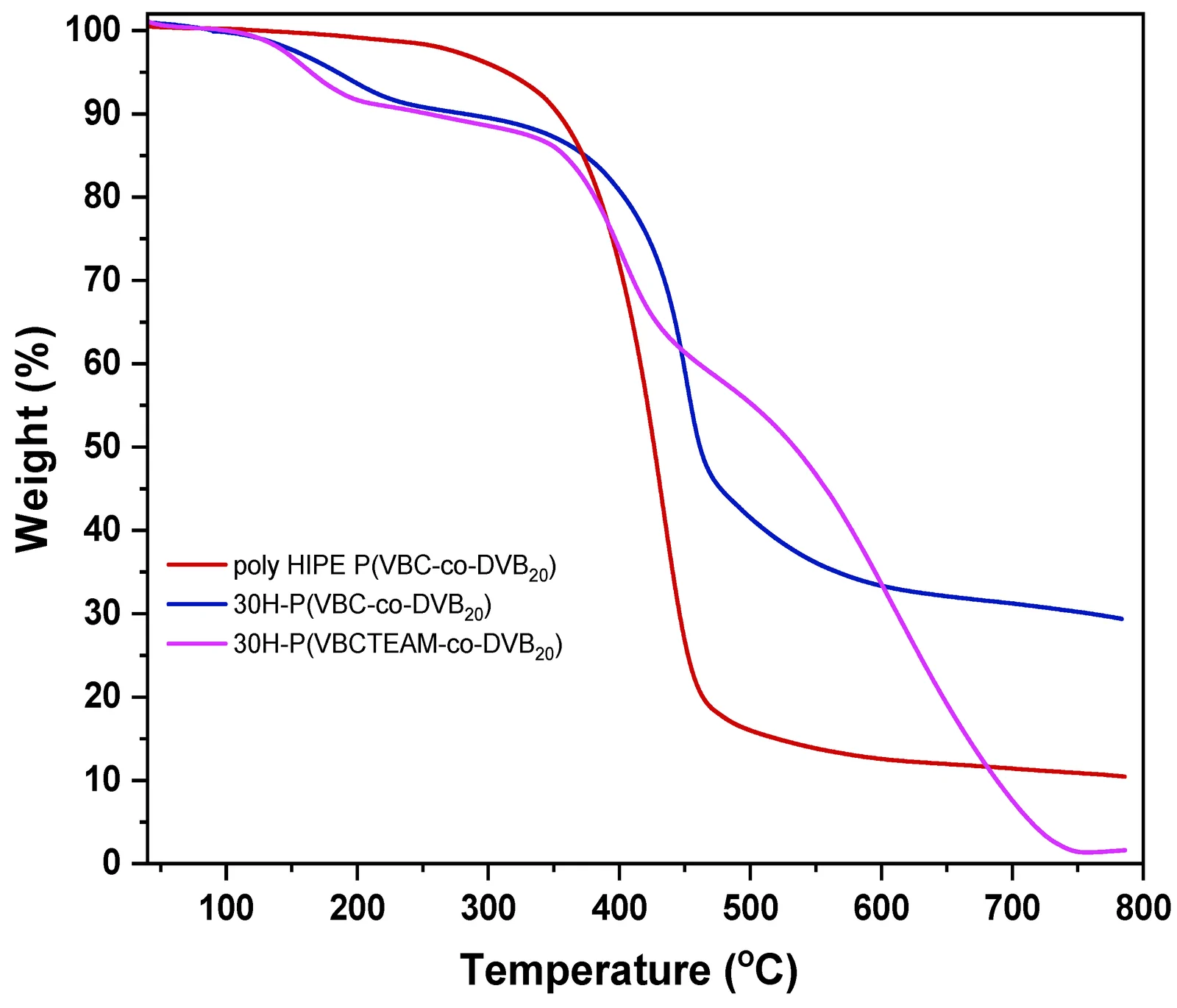
TGA curves of the prepared polyHIPE P(VBC-co-DVB_20_, hypercrosslinked 30H-P(VBC-co-DVB_20_), and its cationic analogue 30H-P(VBCTEAM-co-DVB_20_).

TGA under nitrogen atmosphere was also conducted to study the thermal properties of the prepared polymeric materials (Figure 7). The precursor showed a single-stage weight loss, while a two-stage and a three-stage weight loss were observed for the hypercrosslinked 30H-P(VBC-co-DVB_20_) and its cationic analogue, respectively.

Both hypercrosslinked 30H-P(VBC-co-DVB_20_) and its cationic analogue showed improved thermal stability compared to the precursor, retaining 48–59% and 19% of their initial weight, respectively, at 470 °C. In addition, both samples exhibited an initial weight loss at 120 °C, ascribed to the evaporation of water and residual solvent. In the case of the cationic analogue, the second weight loss between 330 °C and 430 °C is assigned to the degradation of ammonium groups [41], while the third weight loss (>450 °C) corresponds to the degradation of the polymeric backbone.

### 3.2. Adsorption Studies

The interconnected, porous framework of hypercrosslinked PolYHIPE possessing hierarchically macro- and mesopores functions as an attractive substrate for dye adsorption through diffusion within the voids, windows, and mesopores with a pore size ranging between 2 and 50 nm. Moreover, the synthesized cationic hypercrosslinked polyHIPE provides binding sites for the adsorption of anionic dyes through electrostatic interactions. To evaluate its adsorption capacity, the anionic azo dye Acid Blue 113 was selected as a model adsorbate. For this study, the material with the highest surface area, namely 30H-P(VBC-co-DVB_20_), was evaluated and compared to the respective cationic counterpart, 30H-P(VBCTEAM-co-DVB_20_).

**Figure 8:**
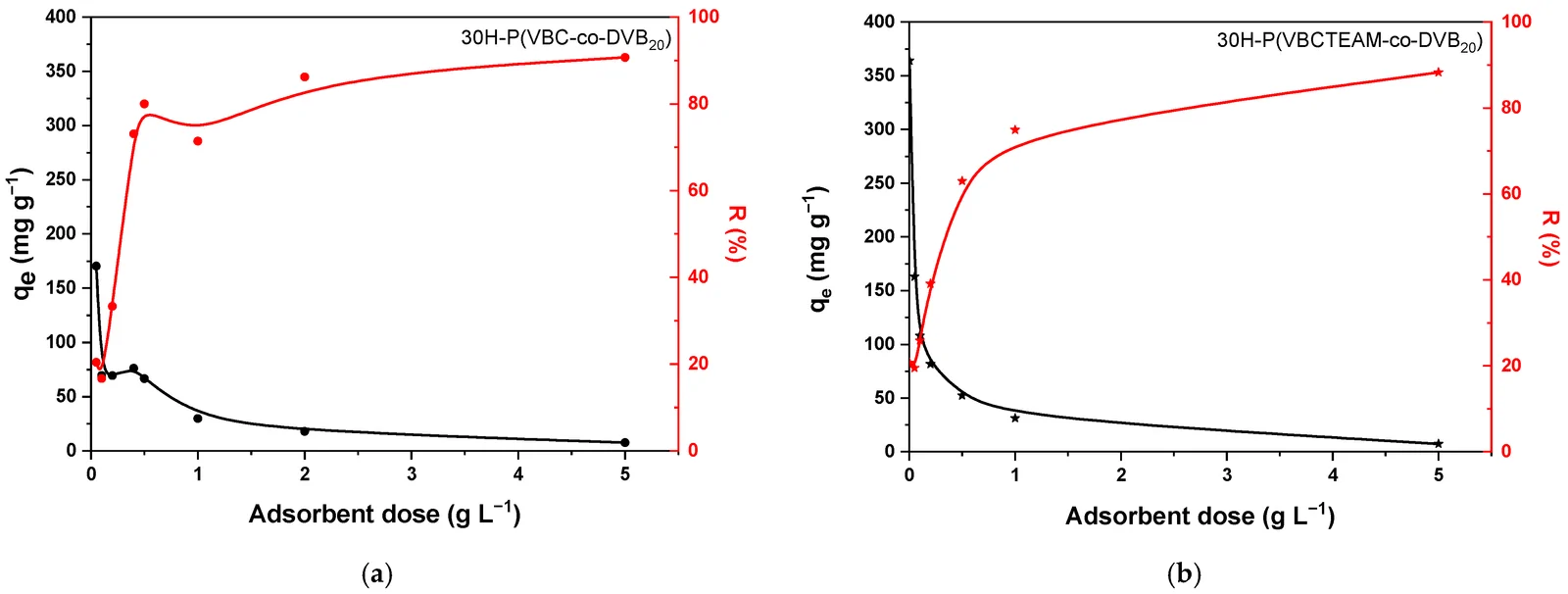
Effect of (**a**) 30H-P(VBC-co-DVB_20_) and (**b**) 30H-P(VBCTEAM-co-DVB_20_) adsorbent dose on the removal efficiency and the adsorption capacity of Acid Blue 113 (C_0_ = 40 mg L^−1^, contact time: 24 h).

The equilibrium adsorption capacity (q_e_) and removal efficiency (R%) of Acid Blue 113 using the two adsorbents 30H-P(VBC-co-DVB_20_) and 30H-P(VBCTEAM-co-DVB_20_) as a function of adsorbent dosage are given in Figure 8. For both adsorbents, a sharp increase in removal efficiency is observed with increasing adsorbent concentration up to ~1 g L^−1^, while the removal efficiency levels off, reaching a value of ~90% at higher adsorbent concentrations. As expected, the trend of q_e_ is reversed within the same adsorbent concentration range. These results are in line with the high availability of active adsorption sites on the adsorbent surface for these adsorbent concentrations (>1g L^−1^). Moreover, the similar high removal efficiencies observed for both materials indicate that 30H-P(VBC-co-DVB_20_) already has sufficient adsorption sites, even before cationic modification.

**Figure 9:**
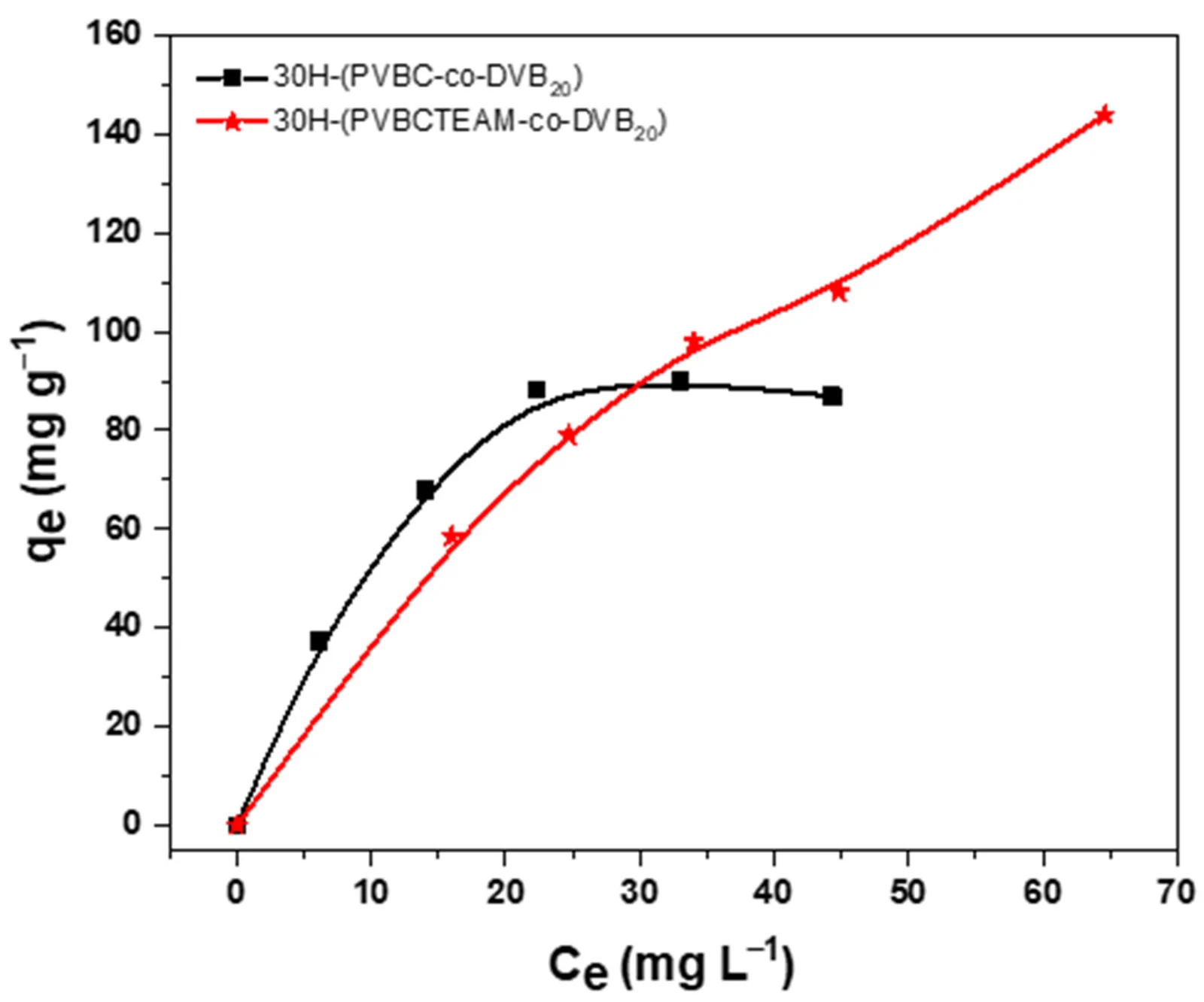
Adsorption isotherms of Acid Blue 113 onto 30H-P(VBC-co-DVB_20_) (black line) and 30H-P(VBCTEAM-co-DVB_20_) (red line) (m = 0.1 g L^−1^, Contact Time: 24 h).

To better elucidate the adsorption behaviour, the adsorption isotherms at 25 °C were constructed for these two systems (Figure 9). In the case of the unmodified hypercrosslinked system, 30H-P(VBC-co-DVB_20_), after an initial increase, the equilibrium adsorption capacity q_e_ seems to reach a plateau value of ~95 mg g^−1^ for equilibrium dye concentrations C_e_ higher than 20 mg L^−1^. In contrast, when the cationic material is used, 30H-P(VBCTEAM-co-DVB_20_), no plateau is observed within the C_e_ range investigated, while q_e_ reaches much higher values for high C_e_. This behaviour indicates that the maximum adsorption capacity of the latter material is much higher and possibly governed by an alternative adsorption mechanism, compared to that of the unmodified precursor.

**Figure 10:**
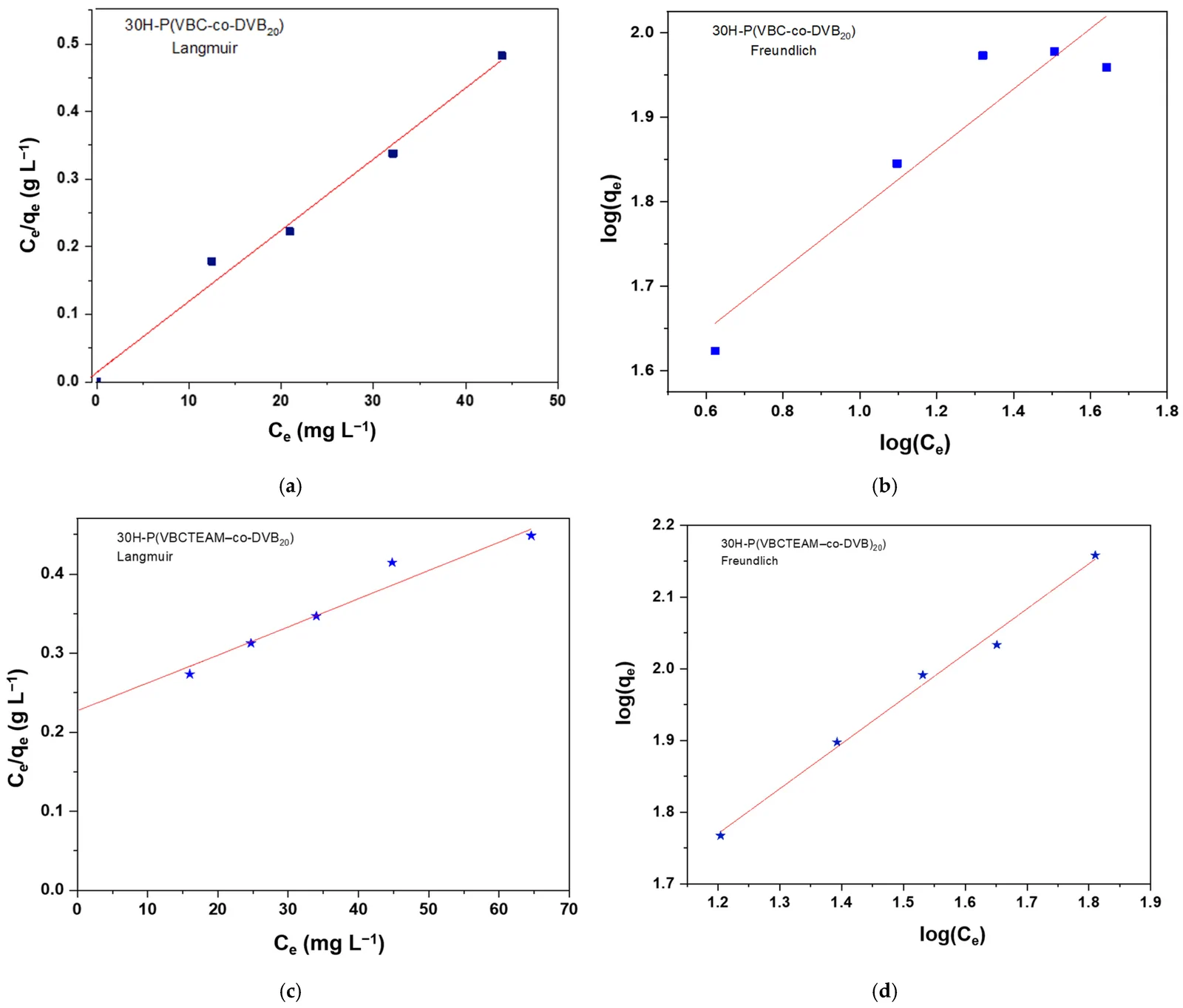
Application of the Langmuir (**a**,**c**) and Freundlich (**b**,**d**) models to 30H-P(VBC-co-DVB_20_) and 30H-P(VBCTEAM-co-DVB_20_) for Acid Blue 113.

The Langmuir isotherm model (Equation (7)) assumes monolayer adsorption on a homogeneous adsorbent surface, whereas the Freundlich model (Equation (9)) describes multilayer adsorption on heterogeneous surfaces. To elucidate the adsorption mechanism of Acid Blue 113, both models were applied to the experimental data (Figure 10 and Table 3). As shown in Table 3, the adsorption of Acid Blue 113 onto 30H-P(VBC-co-DVB_20_) is better described by the Langmuir model, displaying a high correlation coefficient (R^2^ = 0.99), suggesting monolayer adsorption. The maximum adsorption capacity (q) for monolayer coverage was determined to be 110 mg g^−1^ (Table 3). Additionally, the R_L_ (Equation (8)) and 1/*n* values (0 < R_L_ < 1 and 1/*n* < 1) confirm the favourable nature of the adsorption process. In the case of the cationic analogue 30H-P(VBCTEAM-co-DVB_20_), the Langmuir-derived maximum adsorption capacity (q) was found to be much higher, namely 280.1 mg g^−1^ (Table 3). However, the Freundlich model provides a better fit (higher R^2^ values) in this case, indicating possibly a multilayer adsorption mechanism. The R_L_ and 1/*n* values further support the favorability of the adsorption process under all conditions. These results highlight the distinct adsorption behaviour of Acid Blue 113 on the two polymers: monolayer adsorption on 30H-P(VBC-co-DVB_20_) following the Langmuir model and multilayer adsorption on 30H-P(VBCTEAM-co-DVB_20_) following the Freundlich model.

The maximum adsorption capacities, q, of the adsorbents of the present study towards Acid Blue 113 are compared in Table 4 with the respective results reported for other synthetic or natural adsorbents. It is evident that the prepared adsorbents, especially the cationic ones, are well compared with commonly used adsorbent materials reported in the literature.

**Figure 11:**
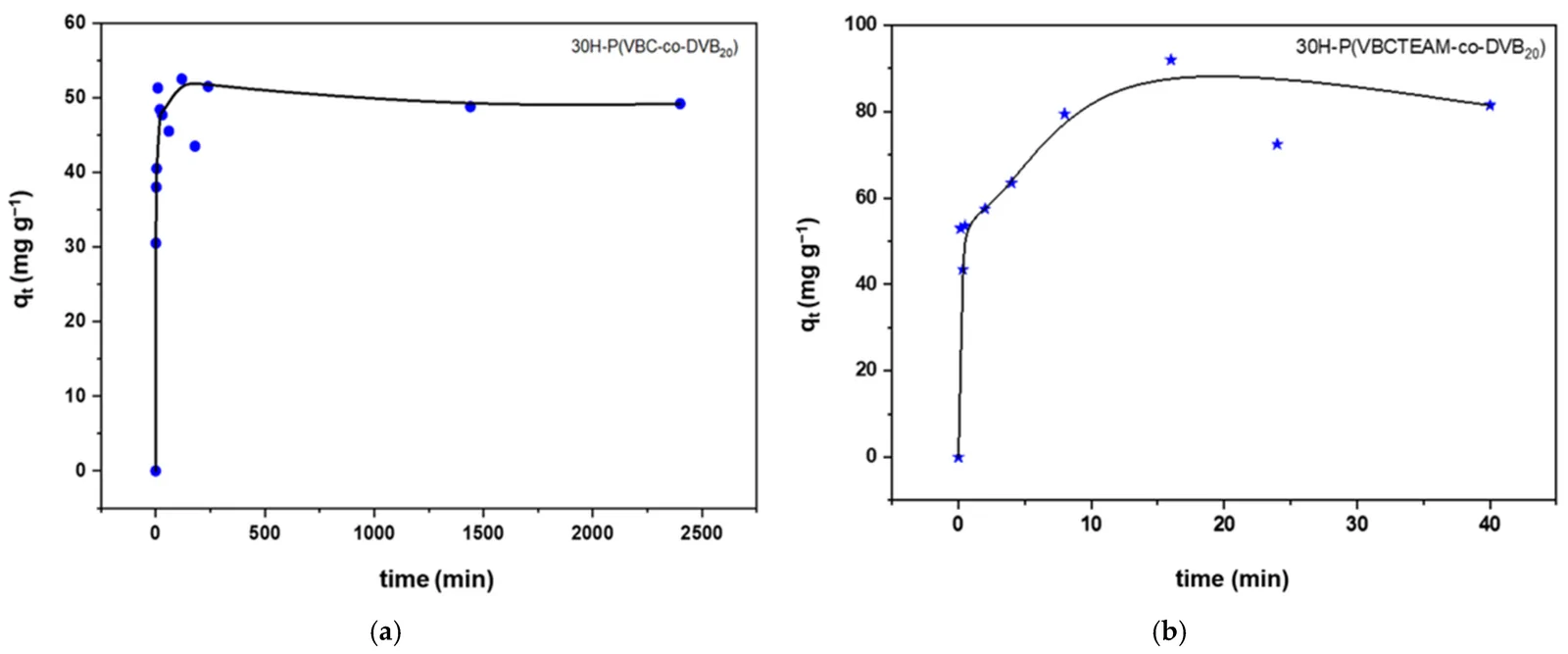
Effect of contact time on the adsorption of Acid Blue 113 onto (**a**) 30H-P(VBC-co-DVB_20_) and (**b**) 30H-P(VBCTEAM-co-DVB_20_).

The effect of contact time on the adsorption capacity of 30H-P(VBC-co-DVB_20_) for Acid Blue 113 was investigated using an initial dye concentration of C_0_ = 40 mg L^−1^ and an adsorbent dosage of 0.2 g L^−1^. The contact time was studied for up to 40 h. As observed in the corresponding adsorption kinetics diagram (Figure 11a), equilibrium was reached within the first few minutes (7–30 min), after which the adsorption capacity (q_t_) did not exhibit a further significant increase. The adsorption capacity for Acid Blue 113 reached 52.5 mg g^−1^ within 2 h. Regarding the cationic analogue, 30H-P(VBCTEAM-co-DVB_20_) experiences an initial sharp increase within the first ~30 min, followed by a second, more gradual increase observed within a few hours (Figure 11b). Equilibrium is achieved after 16 h, under the same conditions, while the adsorption capacity at equilibrium is ~80 mg g^−1^. These findings suggest that there are differences in adsorption kinetics and interaction mechanisms when the two polymeric adsorbents are compared.

**Figure 12:**
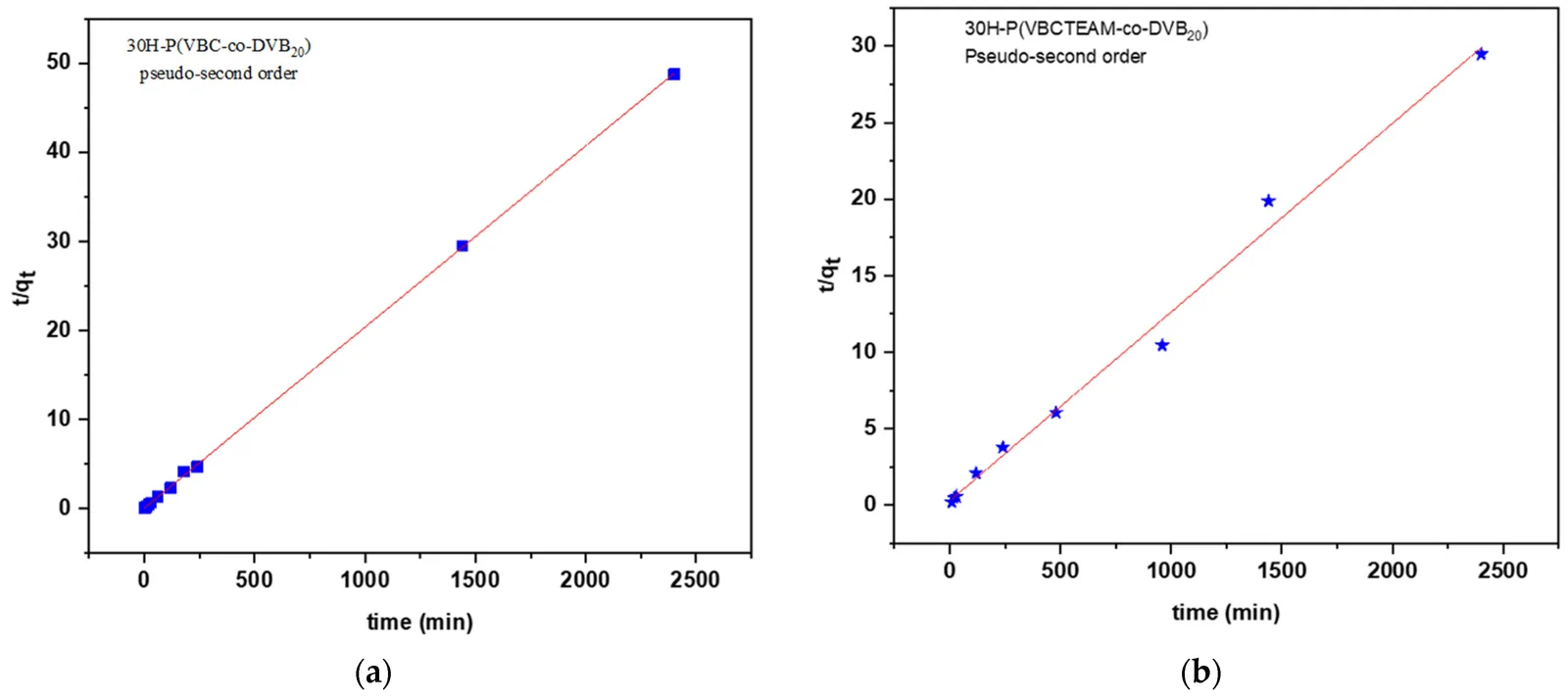
Application of the pseudo-second order model to (**a**) 30H-P(VBC-co-DVB20) and (**b**) 30H-P(VBCTEAM-co-DVB_20_) for Acid Blue 113 dye.

As charge and porous structure are the major contributors to the adsorption capacity, two kinetic models were applied and studied (Figure S2 and Figure 12). The pseudo-first-order model (Equation (4)) was employed; however, as shown in Figure S2a,b, this model did not yield an adequate fit; the calculated adsorption capacity values (q_e,cal_) deviated significantly from the experimental data (q_e,exp_), and the correlation coefficient (R^2^) was unsatisfactory. In contrast, the pseudo-second-order model (Equation (6)) yielded a remarkably better fit. The linear correlation coefficient was quite satisfactory (R^2^ = 0.99, Table 5), and the q_e,cal_ values closely approximated the experimental q_e,exp_, as depicted in Figure 11a,b.

#### Adsorption Mechanism

**Figure 13:**
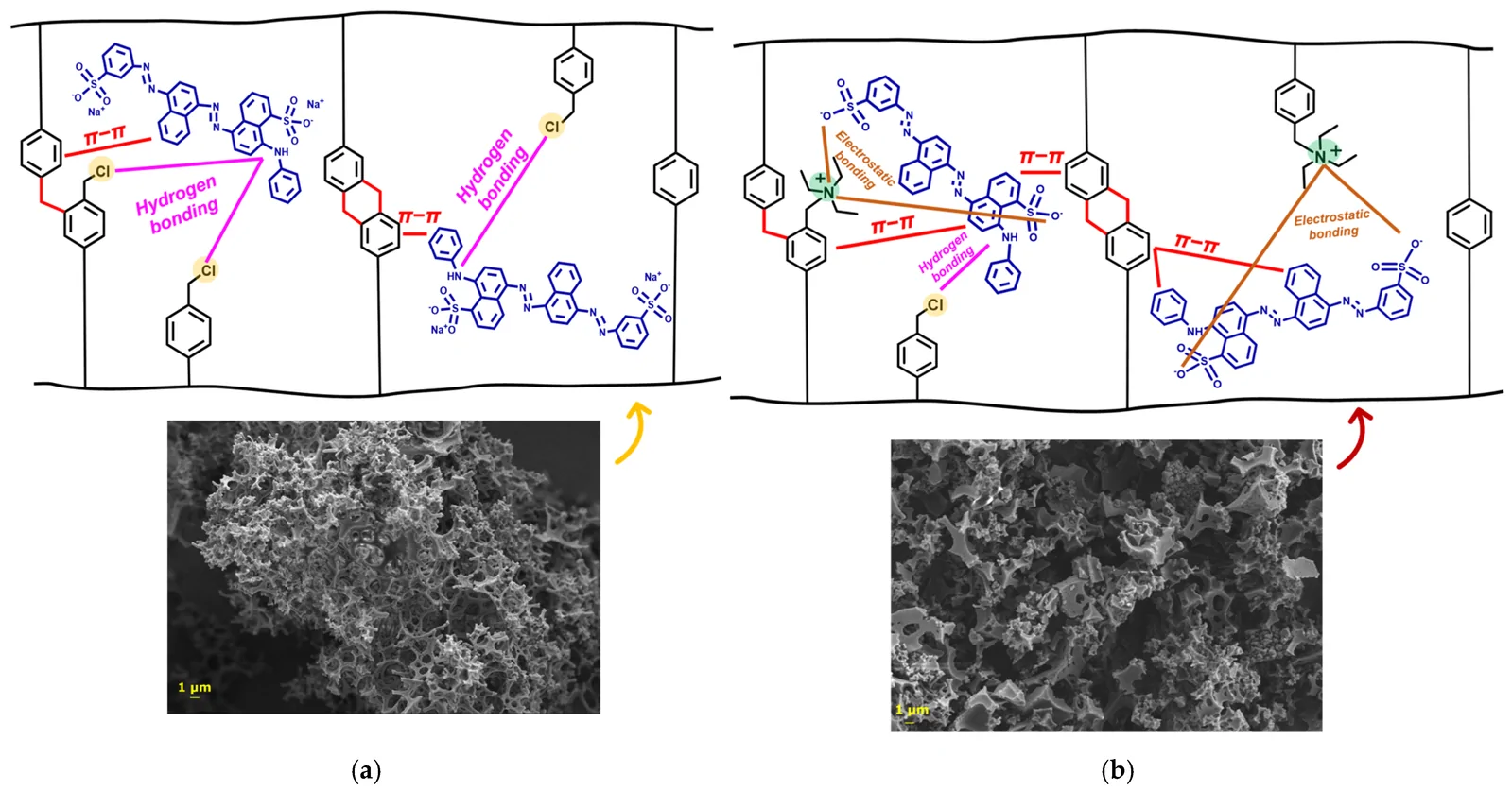
Schematic representation of the adsorption mechanism of anionic Acid Blue 113 dye and the corresponding SEM micrographs after adsorption using (**a**) 30H-P(VBC-co-DVB_20_) and (**b**) 30H-P(VBCTEAM-co-DVB_20_) as adsorbents.

The potential interaction mechanisms upon adsorption of the anionic dye Acid Blue 113 by the hypercrosslinked polymeric adsorbents (30H-P(VBC-co-DVB_20_) and 30H-P(VBCTEAM-co-DVB_20_)) are depicted in Figure 13. Kinetic studies confirmed that adsorption in both systems follows a pseudo-second-order model, suggesting chemisorption as the dominant mechanism. The 30H-P(VBC-co-DVB_20_) adsorbent contains active -CH_2_Cl sites, which are likely to interact with the secondary amine group (-NH) of Acid Blue 113 via hydrogen bonding. Additionally, π–π interactions between the aromatic systems of both adsorbents and the dye further contribute to the adsorption process. In addition to the previous interactions, the cationic adsorbent 30H-P(VBCTEAM-co-DVB_20_), which bears positively charged ammonium (-N^+^(CH_3_)_3_) groups, interacts electrostatically with the two sulfonate (-SO_3_^−^) groups of the dye, thus significantly enhancing the adsorption efficiency. These interaction mechanisms are further supported by the SEM micrographs (Figure 13). A clear modification in the surface morphology of the adsorbents is observed after the adsorption process, which directly indicates the successful incorporation and deposition of the Acid Blue 113 dye onto the surface of both polymeric matrices.

Furthermore, EDS analysis of the adsorbent 30H-P(VBCTEAM-co-DVB_20_) before and after dye adsorption (Figure S3) revealed that after adsorption the Cl content was reduced from 13.8 to 4.9 wt%. This is most likely due to the release in the aqueous solution of Cl^−^ counterions of the cationic adsorbent, caused by the electrostatically driven adsorption of the anionic dye Acid Blue 113. 

**Figure 14:**
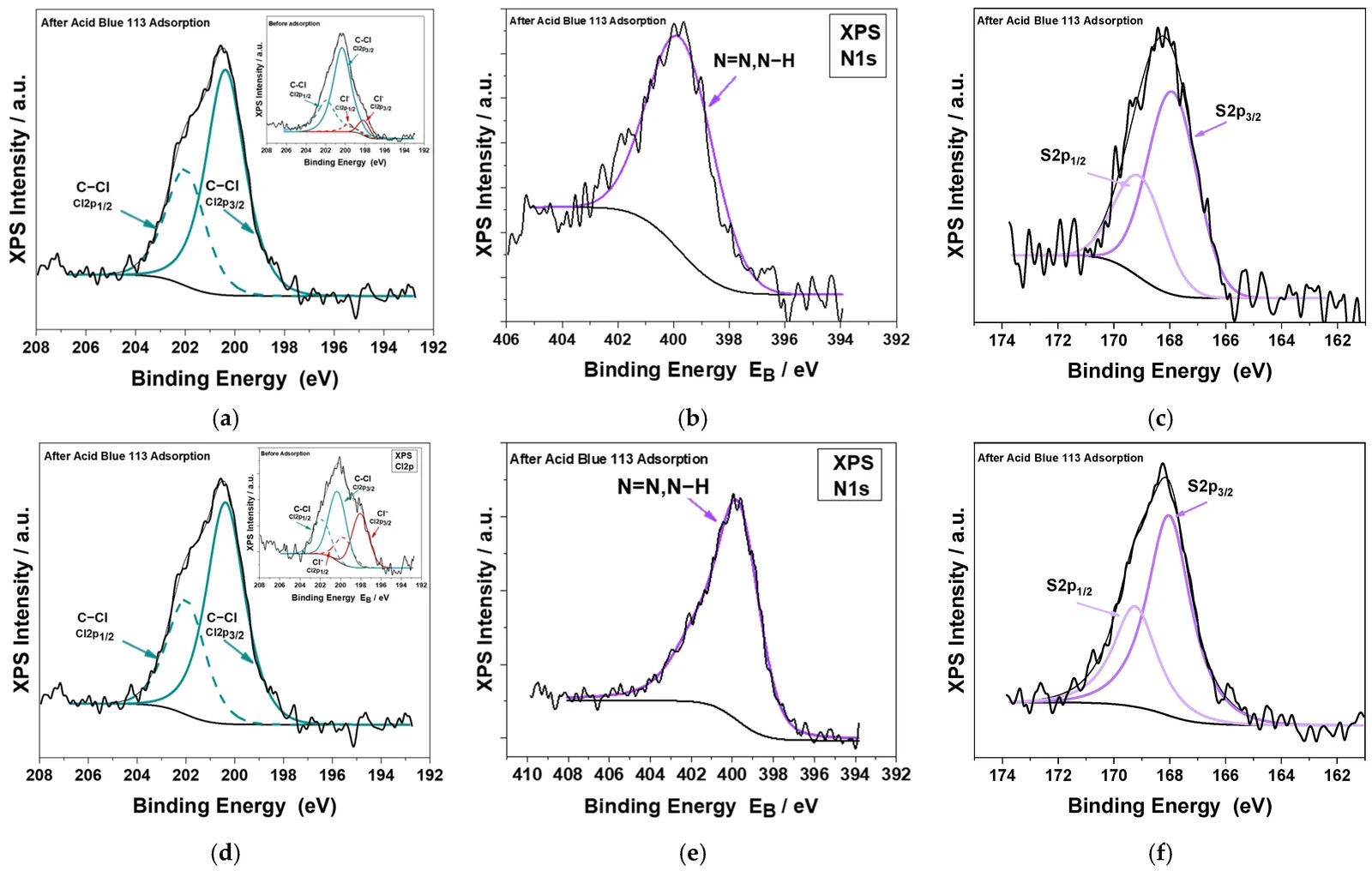
XPS spectra of the prepared (**a**–**c**) 30H-P(VBC-co-DVB_20_) and (**d**–**f**) cationic 30H-P(VBCTEAM-co-DVB_20_) as adsorbents after adsorption of Acid Blue 113 dye.

To gain more insight into the adsorption mechanism of Acid Blue 113, the XPS analysis was performed after dye exhaustion. The successful adsorption of the anionic dye onto both adsorbents was confirmed by the appearance of the S2p doublet in the spectra of both 30H-P(VBC-co-DVB_20_) (Figure 14c) and 30H-P(VBCTEAM-co-DVB_20_) (Figure 14f) located at 168 eV, which is attributed to the sulfonate groups (-SO_3_) of the dye [46]. It is important to mention that, in the Cl2p spectra of both exhausted adsorbents (Figure 14a,d), the peak corresponding to the counterion Cl^−^ at 197.7 eV disappeared completely. At the same time, in the N1s spectrum of the cationic sample, the characteristic N^+^-(CH_2_CH_3_)_3_ component—which had previously been observed at 401.2 eV (Figure 6b)—is absent. The concurrent disappearance of both the counterions Cl^−^ and the distinct N^+^-C peak provides strong evidence for a strong electrostatic interaction and an ion-exchange mechanism between the quaternary ammonium sites of the polymer and the sulfonic groups of the dye.

Furthermore, the N1s spectra after adsorption (Figure 14b,e) were characterized primarily by a peak at 399.7 eV, which corresponds to the azo (-N=N-) and amine groups (N-H) of the adsorbed Acid Blue 113 molecules.

Finally, ethanol-based regeneration cycles of both adsorbents were carried out, resulting in relatively moderate removal efficiency, ascribed to its insufficient ability to disrupt ionic interactions and hydrogen bonding between adsorbents and dye. In addition, upon regeneration cycles, the dye removal efficiency was reduced, which can probably be attributed to the mass loss observed due to structural integrity deterioration of adsorbents. Therefore, this regeneration process raised limitations, and further study for optimization of the procedure is required.

## 4. Conclusions

The development of cationic hypercrosslinked polyHIPEs based on VBC has been reported in the present work. After the preparation of the initial monolith from a water-in-oil (w/o) high internal phase emulsion (HIPE), hypercrosslinking and cationic functionalization were performed, taking advantage of the Friedel–Crafts alkylation reaction and the reaction of the remaining methylene chloride groups with triethylamine (TEAM). These procedures led to materials with high surface area (SBET ~450–600 m^2^ g^−1^) and hierarchical porous structure, as supported by SEM and BET analysis along with cationic functionalities. Because of this structure, the final material has been proven to be an efficient adsorbent of the anionic dye Acid Blue 113, exhibiting a large adsorption capacity of 280 mg g^−1^. The mechanism of the adsorption was chemisorption, as confirmed by kinetic studies. Hydrogen bonding, π-π interactions, and mainly electrostatic interactions between ammonium groups of cationic hypercrosslinked polyHIPE and sulfonate groups of the dye contributed to the adsorption process, as revealed by XPS.

These findings suggest that cationic hypercrosslinked VBC-based HIPEs are promising materials for wastewater remediation. Moreover, the reactivity of benzyl chloride towards a variety of chemical species enables the versatile design of hypercrosslinked HIPEs with desirable chemistry and functions.

## Figures and Tables

**Table 1:** HIPE composition.

Sample		P(VBC-co-DVB_20_)	
Organic External Phase *w*/*w*%		Aqueous Internal Phase *w*/*w*%	
4-VBC	8.3	H_2_O	87
DVB	1.7	K_2_S_2_O_8_	0.2
Span 80	2.9	CaCl_2_	0.4
Total	12.9	Total	87.6

**Table 2:** Surface area and porosity parameters of polyHIPE P(VBC-co-DVB_20_) and hypercrosslinked polyHIPEs and their corresponding cationic analogue.

Sample	S_BET_ (m^2^ g^−1^) ^a^	V_total_ (cm^3^ g^−1^) ^b^	D_ave_ (nm) ^c^
polyHIPE P(VBC-co-DVB_20_)	10.1	0.02	15
30H-P(VBC-co-DVB_20_)	614.5	0.36	5.4
60H-P(VBC-co-DVB_20_)	534.7	0.31	5.3
180H-P(VBC-co-DVB_20_)	461.6	0.27	4.6
30H-P(VBCTEAM-co-DVB_20_)	566.9	0.32	5.6

^a^ BET surface area. ^b^ total pore volume. ^c^ average pore size.

**Table 3:** Estimated parameter values and correlation coefficients of Langmuir and Freundlich models for the adsorption of Acid Blue 113 on the 30H-P(VBC-co-DVB_20_) and 30H-P(VBCTEAM-co-DVB_20_).

	30H-P(VBC-co-DVB_20_)	30H-P(VBCTEAM-co-DVB_20_)
Parameters	Acid Blue 113	
	Langmuir	
q (mg g^−1^)	110	280.1
K_L_ (L mg^−1^)	0.11	0.0158
R_L_	0.1453–0.479	0.445–0.7434
R^2^	0.968	0.961
	Freundlich	
K_F_ (mg g^−1^) (L mg^−1^)^1/n^	19	10.4
1/*n*	0.444	1.017
R^2^	0.862	0.992

**Table 4:** Comparison of the maximum adsorption capacity of 30H-P(VBC-co-DVB_20_) and 30H-P(VBCTEAM-co-DVB_20_) with the respective behaviour of other adsorbents towards Acid Blue 113, reported in the literature.

Adsorbents	q (mg g^−1^)	Reference
Chitosan-Fe_2_O_3_	128.2	[42]
Chitin Chitosan	125 90.9	[43]
P4VP-C16Br (Poly(N-hexadecyl-4-vinylpyridinium bromide)	204.4	[44]
Amberlite IRA 402(Cl-) resin	117	[45]
30H-P(VBC-co-DVB_20_)	**110**	**This work**
30H-P(VBCTEAM-co-DVB_20_)	**280.1**	**This work**

**Table 5:** Kinetic parameters for Acid Blue 113 adsorption on 30H-P(VBC-co-DVB_20_) and 30H-P(VBCTEAM-co-DVB_20_).

	30H-P(VBC-co-DVB_20_)	30H-P(VBCTEAM-co-DVB_20_)
Parameters	Acid Blue 113^(40h)^	
q_e,exp_ (mg g^−1^)	52.5	79.5
	**Pseudo-first order**	
q_e,cal_ (mg g^−1^)	7.03	26.8
k_1_ × 10^−3^ (1 min^−1^)	0.346	0.966
R^2^	0.11	0.89
	**Pseudo-second order**	
q_e,cal_ (mg g^−1^)	49.2	81.04
k_2_ × 10^−5^ (g mg^−1^ min^−1^)	0.079	0.01
R^2^	0.99	0.99

## Data Availability

The original contributions presented in this study are included in the article/Supplementary Materials. Further inquiries can be directed to the corresponding author.

## Supplementary Materials

The following supporting information can be downloaded at: https://www.mdpi.com/article/10.3390/ma19173680/s1, Figure S1: Calibration curve of Acid Blue 113; Figure S2: Application of the pseudo–first order (a,b) model to 30H-P(VBC-co-DVB_20_) and 30H-P(VBCTEAM-co-DVB_20_) for Acid Blue 113 dye; Figure S3: EDS analysis of 30H-P(VBCTEAM-co-DVB_20_) before (a) and (b) after adsorption of Acid Blue 113 dye.
