# Supplementary material for: Functionalized Hypercrosslinked High Internal Phase Emulsion Materials Based on 4-Vinylbenzyl Chloride for Anionic Dye Adsorption
Source: Materials (Basel). 2026 Aug 29;19(17):3680. doi: 10.3390/ma19173680 (PMC13567699; doi:10.3390/ma19173680)
Supplement: Supplementary file 1 [file materials-19-03680-s001.zip › materials-4487945-supplementary.pdf]

Supplementary Materials

Functionalized Hypercrosslinked High Internal Phase Emulsion Materials Based on 4-Vinylbenzyl Chloride for Anionic Dye Adsorption

Eftychia Tsolka [1], Labrini Sygellou [2], Georgios Bokias [1,2] and Valadoula Deimede [1,*]

[1] Department of Chemistry, University of Patras, GR-26504 Patras, Greece; efi.tsolka@gmail.com (E.T.); bokias@upatras.gr (G.B.)
[2] Foundation for Research and Technology—Hellas (FORTH), Institute of Chemical Engineering Science (ICE-HT), Stadiou St., GR-26504 Patras, Greece; sygellou@iceht.forth.gr
* Correspondence: deimede@upatras.gr; Tel.: +30-2610-962958:

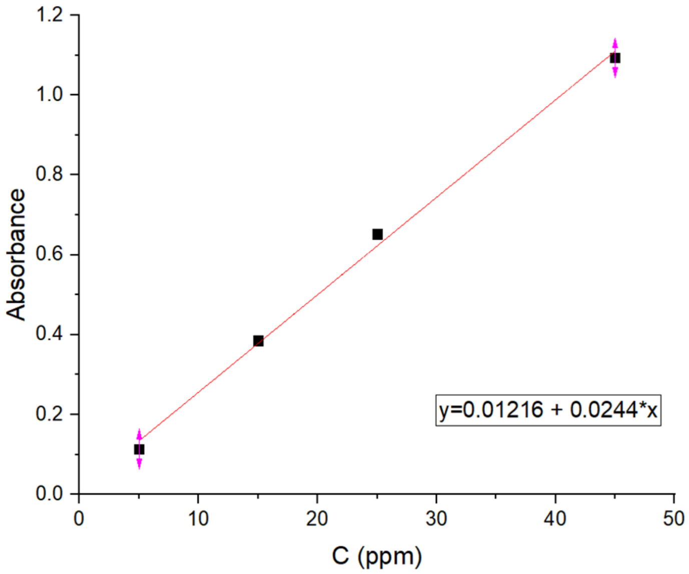

Figure S1. Calibration curve of Acid Blue 113.

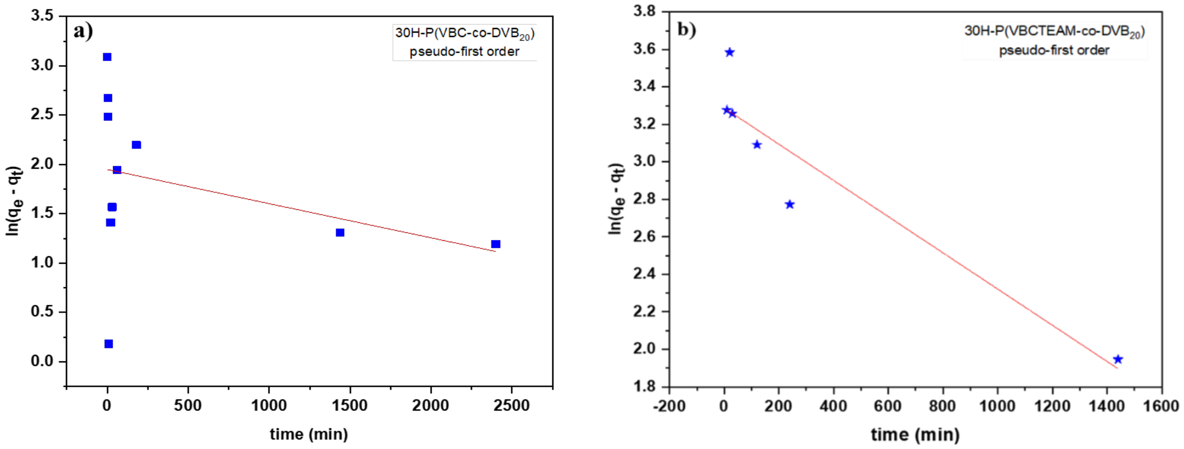

Figure S2. Application of the pseudo-first-order model to (**a**) 30H-P(VBC-co-DVB$_{20}$) and (**b**) 30H-P(VBCTEAM-co-DVB$_{20}$) for Acid Blue 113 dye.

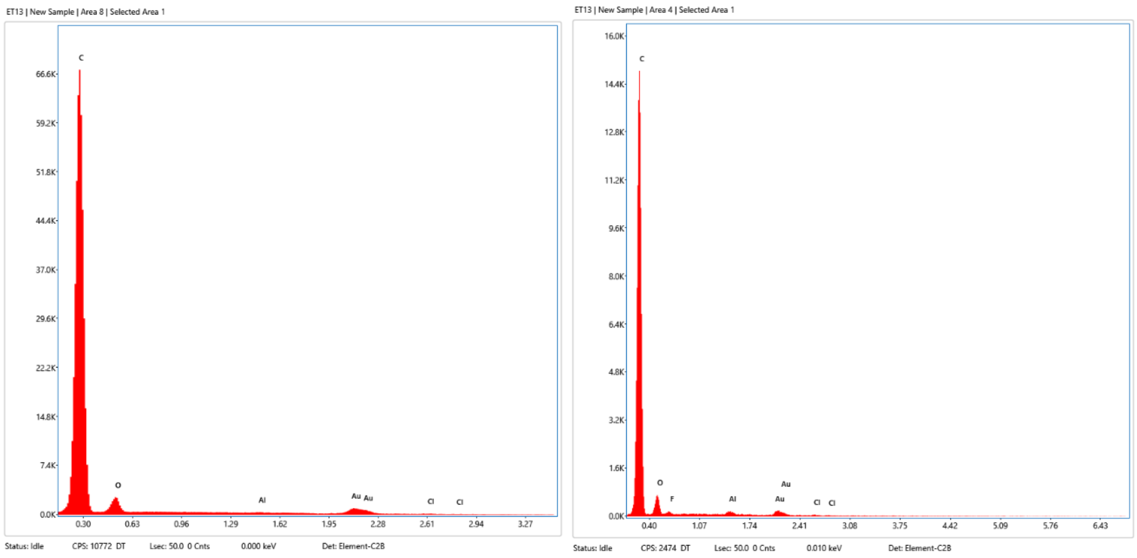

(**a**) (**b**)

Figure S3. EDS analysis of 30H-P(VBCTEAM-co-DVB$_{20}$) before (**a**) and after (**b**) adsorption of Acid Blue 113 dye.
